# A Clinical Prediction Score in Addition to WHO Criteria for Anti-Retroviral Treatment Failure in Resource-Limited Settings - Experience from Lesotho

**DOI:** 10.1371/journal.pone.0047937

**Published:** 2012-10-31

**Authors:** Niklaus Daniel Labhardt, Thabo Lejone, Matse'liso Setoko, Matalenyane Poka, Jochen Ehmer, Karolin Pfeiffer, Patrice Zinga Kiuvu, Lutgarde Lynen

**Affiliations:** 1 SolidarMed Lesotho, Seboche Hospital, Botha-Bothe, Lesotho; 2 Seboche Hospital, Botha-Bothe, Lesotho; 3 Lesotho Central Laboratory, Maseru, Lesotho; 4 SolidarMed Switzerland, Lucerne, Switzerland; 5 Department of Clinical Sciences, Institute of Tropical Medicine, Antwerp, Belgium; University of Pittsburgh, United States of America

## Abstract

**Objective:**

To assess the positive predictive value (PPV) of a clinical score for viral failure among patients fulfilling the WHO-criteria for anti-retroviral treatment (ART) failure in rural Lesotho.

**Methods:**

Patients fulfilling clinical and/or immunological WHO failure-criteria were enrolled. The score includes the following predictors: Prior ART exposure (1 point), CD4-count below baseline (1), 25% and 50% drop from peak CD4-count (1 and 2), hemoglobin drop≥1 g/dL (1), CD4 count<100/µl after 12 months (1), new onset papular pruritic eruption (1), and adherence<95% (3). A nurse assessed the score the day blood was drawn for viral load (VL). Reported confidence intervals (CI) were calculated using Wilsons method.

**Results:**

Among 1'131 patients on ART≥6 months, 134 (11.8%) had immunological and/or clinical failure, 104 (78%) had blood drawn (13 died, 10 lost to follow-up, 7 did not show up). From 92 (88%) a result could be obtained (2 samples hemolysed, 10 lost). Out of these 92 patients 47 (51%) had viral failure (≥5000 copies), 27 (29%) viral suppression (<40) and 18 (20%) intermediate viremia (40–4999). Overall, 20 (22%) had a score≥5. A score≥5 had a PPV of 100% to detect a VL>40 copies (95%CI: 84–100), and of 90% to detect a VL≥5000 copies (70–97). Within the score, adherence<95%, CD4-count<100/µl and papular pruritic eruption were the strongest single predictors. Among 47 patients failing, 8 (17%) died before or within 4 weeks after being switched. Overall mortality was 4 (20%) among those with score≥5 and 4 (5%) if score<5 (OR 4.3; 95%CI: 0.96–18.84, p = 0.057).

**Conclusion:**

A score≥5 among patients fulfilling WHO-criteria had a PPV of 100% for a detectable VL and 90% for viral failure. In settings without regular access to VL-testing, this PPV may be considered high enough to switch this patient-group to second-line treatment without confirmatory VL-test.

## Introduction

Identifying patients failing on first-line treatment is a major challenge in anti-retroviral treatment (ART) programs in resource-limited settings. As a result patients who are failing on a first-line regimen are often not or not timely switched to a second-line regimen [1]. This leads to a high mortality among patients who fail on first-line ART [1]–[3]. The World Health Organization's (WHO) clinical and immunological failure-criteria showed only limited accuracy. Various studies report a positive predictive value (PPV) of the WHO-criteria for viral failure ranging from 6% to 39% [4], [5]. Thus a confirmatory viral load (VL) measurement is recommended, before any switch to second-line can be considered [5]–[8]. However, in many settings access to viral load testing is difficult and is causing delays in treatment switch.

Lynen et al. published in 2009 a clinical predictor score to identify patients in need for targeted VL testing based on the score's post-test probability for viral failure [9].

The score is based on reported adherence, prior ART exposure, clinical observation of papular pruritic eruption, and trends in CD4-count and haemoglobin-level.

They recommended that only patients with a score 2–4 needed VL testing, as the post-test probability for viral failure in individuals with a score<2 was very low and in case of ≥5 very high. However, the score was derived from a cohort in Cambodia, where only three patients had a score≥5. In a first validation study conducted in Uganda, only 1 patient had a score≥5 [10].

The objective of this study is to assess the PPV of a score≥5 among patients fulfilling the WHO-criteria for treatment failure in a rural cohort of Lesotho, with the aim to reduce the delays in switching to second-line treatment when needed.

## Methods

### Ethics Statement

The study protocol was approved by the Ethical Committee of the Ministry of Health and Social Welfare of Lesotho. All patients gave oral and written consent. In the case of children <16 years of age, the care-taker gave oral and written consent.

### Study objectives and design

The primary objective of this cross-sectional study was to determine the PPV of a clinical score for viral failure among patients on first-line ART who fulfill the clinical and/or immunological WHO-criteria for treatment failure in rural Lesotho. Secondary objectives were to assess the overall failure-rate among patients fulfilling the WHO-criteria, the PPV of each single predictor within the score and the mortality among patients with confirmed viral failure before or within 4 weeks after switch to second-line ART.

### Study setting

The study was conducted between October 2010 and April 2011 in the catchment area of Seboche Hospital in northern Lesotho. Seboche Hospital and its 5 affiliated nurse-led health centers serve an estimated catchment population of 55’000. The whole catchment area benefits from support of SolidarMed, a non-governmental organization based in Switzerland who supported the provision of ART since 2005. In October 2010, 1’301 patients were on ART. Of these, 691 (53%) were followed at one of the five health centers, the remaining at the hospital. ART is available free of costs at health facilities in Lesotho. Clinical monitoring is usually done on monthly basis, CD4 counts are determined every 6 months. In case of suspected treatment failure, clinicians have to write a second-line request to a second-line committee headed by the Ministry of Health and Social Welfare of Lesotho. The committee replies within one to two months. Usually, for the request to be approved, confirmatory VL testing is required. At the time the study was conducted, no VL testing was available within Lesotho. However, in case of immunological or clinical failure, the Ministry provides funds for a limited number of VL tests to be performed in a laboratory in South Africa. For this purpose, blood samples have to arrive before 2 pm at the central laboratory in Maseru in order to be further transported to South Africa. All VL obtained in this study followed this procedure.

### Study procedure and selection of participants

All patients on first-line ART since at least 6 months, aged ≥10 years, who fulfilled clinical and/or immunological WHO-criteria for treatment failure and who were followed within the study area, were eligible for the study. Clinical failure was defined as a new or recurrent WHO-stage 4 event after at least 6 months of ART. Immunological failure was defined as a drop of the CD4 count back to or below baseline or a ≥50% drop of CD4-count from the on-treatment CD4-peak or persistent CD4 levels <100 cells/µl after ≥12 months on treatment [11]. Even though the second and third immunological WHO-criteria are only applicable for patients on ART since at least 12 months, patients who were on ART for less than 12 months who showed a new clinical stage 4 event after ≥6 months on ART or whose CD4-count fell below pre-treatment levels were included in the study. Patients taking protease-inhibitor based ART were excluded from the study. ART-nurses at hospital and health centers were trained to assess the WHO-criteria for treatment failure and went through all patient-records checking for immunological or clinical signs of failure to generate a list of eligible patients. All eligible patients were scheduled on pre-defined days for blood-draw at Seboche Hospital. Blood-samples were transported the same day to the Central Laboratory of Lesotho at Maseru for further processing. Patients not attending the first scheduled visit were traced and given a second appointment. Table 1 displays the components of the score used in this study. The day blood for VL was drawn, a nurse-clinician assessed the clinical part of the score (adherence measured by a visual analogue scale (VAS), prior exposure to HAART, presence of recurrent papular pruritic eruption). At the same moment blood was drawn for VL, a second tube to repeat CD4-count and hemoglobin was taken. This was done to ensure that CD4-counts and hemoglobin levels used to calculate the score were contemporary, as some patients who were eligible based on the records had their last CD4-count several months prior to the day they came for VL-measurement. All patients who participated in the study and who did not show up for their ART follow-up visits after VL was measured were traced to ascertain if they were alive and to encourage their return back to care.

**Table 1:** The clinical score used in this study to predict viral failure.

Predictor	Score if condition is present
**Adherence <95% (using visual analogue scale)**	3
**CD4 count falls below baseline**	1
**>25% CD4 count decrease from on-treatment peak**	1
**>50% CD4 count decrease from on-treatment peak**	1
**CD4 count <100 cells/µl after 12 months of ART**	1
**Prior exposure to HAART** *	1
**Hemoglobin drop ≥1 g/dl** ¶	1
**New onset or recurrent papular pruritic eruption**	1
**Maximum SCORE (Sum)**	**10**

The score was originally developed in a Cambodian cohort [9].

* HAART, Highly Active Antiretroviral Therapy.

¶ Comparing current hemoglobin level to a documented hemoglobin measurement 3 to 9 months ago.

### Data-processing and statistical analysis

All patient-data to build the score were collected the same day the blood sample for VL was taken. Data were entered anonymously using EpiData 3.1® and stored on a password-protected computer. An undetectable VL (<40 copies/µl) was considered as viral suppression, a VL between 40 and 5’000 as indeterminate result and ≥5’000 as viral failure. Based on the clinical score, two subgroups of patients were formed: those with a score<5 and those with a score≥5. The PPV for a VL>40 or a VL≥5’000 copies was calculated as the proportion of individuals with a VL>40 or a VL≥5’000 respectively out of the subgroup of all patients who have a score≥5. Confidence intervals (CI) for the PPV were calculated using Wilsons score method. To assess the risk of mortality among patients continuing first-line ART while waiting for the VL-result and the approval from the MoHSW to be switched to second-line, outcomes of all patients were determined eight weeks after the blood draw for VL. In case of patients with viral failure, their status (dead or alive) was checked the moment they were supposed to start second-line ART (the moment the approval of the MoHSW was received). In patients who were able to start second-line ART at that moment, the status (dead or alive) was again determined 4 weeks after the switch. Risk of mortality during this time-period between blood-draw for VL and 4 weeks after switch to second-line was assessed using univariate logistic regression, with a score≥5 as predictor. All analyses were run on STATA 10.1™.

## Results

### Participants

**Figure 1:**
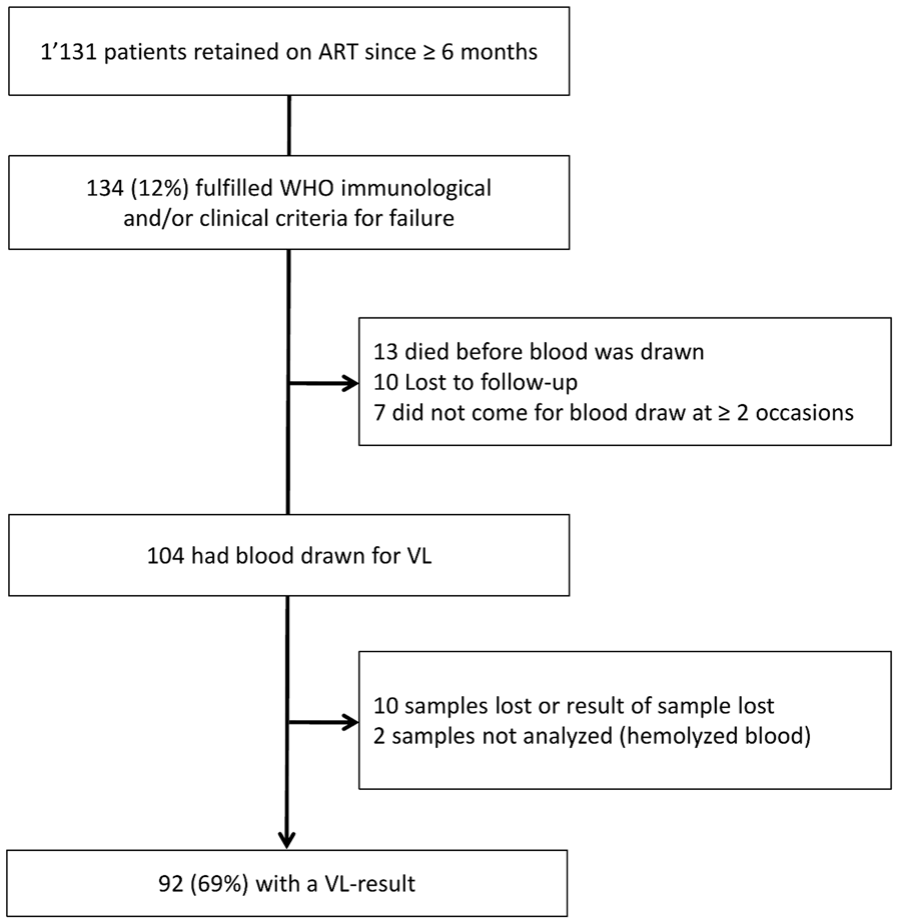
Patient-flow in the study. 92 (69%) of the patients initially eligible for the study received a VL-result and were included in the final analysis. VL: viral load. ART: Anti-retroviral Therapy. WHO: World Health Organization.

Among the 1’301 patients retained and on ART in the study area by October 1^st^ 2010, 1’131 were on ART since at least 6 months, and 134 (11.8%) fulfilled WHO-criteria for immunological and/or clinical failure. Of those, 104 (78%) had blood drawn on one of the scheduled days, 13 had died, 10 were lost to follow-up and 7 did not manage to come at several occasions (see figure 1). From 92 (88%) of the samples a result could be obtained (2 samples hemolysed, 10 were lost during transport/processing in the laboratory). Of the 92 patients enrolled in the study, 75 (82%) had only immunological failure, 1 had only clinical failure and 16 (17%) fulfilled immunological as well as clinical WHO criteria for treatment failure. Most (89) were on ART since at least 12 months. Table 2 shows characteristics of the 92 patients included in the analysis.

**Table 2:** Characteristics of the 92 patients included in the analysis.

Characteristics	n = 92
Female sex	45 (49%)
Median age (iqr)	41 (33–49)
Aged 10–15 years	10 (11%)
Median months on ART (iqr)	29 (19–41)
Previous ART-exposure	
- HAART	4 (4%)
- PMTCT	2 (2%)
Previous interruption of ART≥3 days	17 (18%)
Adherence (VAS) (iqr)	100% (95–100)
NRTI-backbone of current regimen	
- stavudine/lamivudine	12 (13%)
- zidovudine/lamivudine	37 (40%)
- tenofovir/lamivudine	43 (47%)
Previous drug-substitution within first-line	13 (14%)
New onset of papular pruritic eruption	27 (29%)
New WHO stage 3 or 4 event while on ART	17 (19%)
CD4 count the day blood for VL was drawn (cells/µl) (iqr)	127 (70–188)
Hemoglobin the day blood for VL was drawn (g/dl) (iqr)	12.9 (11.4–13.9)
WHO-immunological criteria:	
- Drop of CD4 below baseline	55 (60%)
- CD4 below 100/µl after 1 year on ART	27 (29%)
- ≥25% drop of CD4 from the on-treatment peak	13 (14%)
- ≥50% drop of CD4 from the on-treatment peak	71 (77%)
Number of immunological WHO criteria	
- 0 criteria	1 (1%)
- 1 criteria	37 (40%)
- 2 criteria	46 (50%)
- 3 criteria	8 (9%)

IQR: inter-quartile range. ART: Anti-retroviral therapy. WHO: World Health Organization. HAART: Highly active anti-retroviral therapy. PMTCT: Prevention of mother-to-child transmission.

From the date of blood-draw to the date the VL-result was obtained, a median time of 28 days (inter-quartile range 25–56) elapsed. Among those patients with viral failure, a median time of 55 days (39–74) elapsed between the day they were scheduled for VL blood-draw and the day a response from the second-line committee was obtained.

### Positive Predictive Value of the Clinical Score

Out of the 92 patients with a VL result, 47 (51%) had viral failure (VL≥5000 copies), 27 (29%) viral suppression (<40) and 18 (20%) an intermediate result (40–4999).

Overall, 20 (22%) had a clinical score≥5. A score≥5 had a PPV of 100% to detect a VL>40 copies (95%CI: 84–100), and of 90% to detect a VL≥5000 copies (95%CI: 70–97) (table 3). The 72 patients with a score<5 had still a probability of 40% (95%CI: 30–52) for a VL≥5’000. As shown in table 3, out of the 8 components of the score, an adherence measured by VAS of less than 95%, new onset papular pruritic eruption and a CD4-count <100 cells/µl had the highest positive predictive value as a single predictor in addition to the WHO-criteria. For a detectable VL and viral failure, the PPV of VAS<95% (n = 15) alone was 100% (95%CI: 80–100) and 93% (70–99) respectively, for recurrent papular pruritic eruption (n = 27) it was 89% (72–96) and 85 (68–94) and for a persistent CD4-count below 100cells/µl (n = 27) it was 93% (77–98) and 89% (72–96).

**Table 3:** Positive predictive value of the clinical score and the WHO criteria.

Predictor	N	VL≥40 copies (n = 65)	VL≥5000 copies (n = 47)
**Score**			
- 1	2	1 (50%)	1 (50%)
- 2	17	11 (65%)	4 (24%)
- 3	32	20 (63%)	13 (41%)
- 4	21	13 (62%)	11 (52%)
- 5	6	6 (100%)	4 (67%)
- 6	7	7 (100%)	7 (100%)
- 7	4	4 (100%)	4 (100%)
- 8	3	3 (100%)	3 (100%)
**Score cut-offs**			
Score≥1	92	65 (71%)	47 (51%)
Score≥2	90	64 (71%)	46 (51%)
Score≥3	73	53 (72%)	42 (58%)
Score≥4	41	33 (81%)	29 (71%)
Score≥5	20	20 (100%)	18 (90%)
Score≥6	14	14 (100%)	14 (100%)
**Predictive value of each predictor in the score**			
CD4-count <100 after ≥12 months of ART	27	25 (93%)	24 (89%)
≥25%drop in CD4 while on ART	84	58 (69%)	40 (48%)
≥50% drop in CD4 while on ART	71	48 (68%)	32 (45%)
CD4-count drops back to baseline	55	40 (73%)	28 (51%)
Prior exposure to HAART	4	2(50%)	2(50%)
Hemoglobin-drop ≥1 g/dl	27	17 (63%)	15 (56%)
New or recurrent papular pruritic eruption	27	24 (89%)	23 (85%)
Adherence <95% by VAS	15	15 (100%)	14 (93%)
**Predictive value of WHO-criteria**			
Any criteria	92	65 (71%)	47 (51%)
Immunological criteria only	75	49 (65%)	33 (44%)
Clinical criteria only	1	1 (100%)	1 (100%)
Immunological and clinical criteria	16	15 (94%)	13 (81%)

**Clinical score and WHO-criteria as predictors for a detectable viral load (≥40 copies) or viral failure (≥5’000 copies) among the 92 patients analysed for the study.**

VL: Viral load. WHO: World Health Organization.

### Positive Predictive Value of the WHO-criteria and Mortality

The probability of viral failure (≥5’000 copies) in patients who only had immunological WHO criteria was 44% (95%CI: 33–55) as compared to 81% (95%CI: 57–93) in patients with immunological and clinical WHO criteria combined (table 3). However, patients who had only immunological failure but a score≥5 (n = 13) had viral failure in 92% (95%CI: 67–98) of cases.

Among the 47 patients with viral failure, 8 (17%) died before or within 4 weeks after being switched. Among the patients with VL<5000 all 45 were retained 8 weeks after blood was drawn for VL. The overall mortality among the 92 participants was 4 out of 20 (20%) in the group with score≥5 and 4 out of 72 (5%) among those with a score<5 (OR 4.3; 95%CI: 0.96; 18.84, p = 0.057).

## Discussion

This cross-sectional study assessed the PPV of a clinical score for viral failure among patients on first-line ART who fulfilled the immunological and/or clinical WHO-criteria for treatment failure in rural Lesotho. The score applied in this study was originally derived from a cohort in Cambodia with 764 individuals [9]. We found that a score≥5 had a high PPV for viral failure within a subgroup of patients in rural Lesotho who already fulfilled the immunological and/or clinical WHO-criteria. The PPV was 100% for a detectable viral load and 90% for a VL≥5’000 copies, while for the WHO-criteria alone the PPV was 71% and 51%, respectively (table 3). Moreover, patients with a score≥5 tend to have a higher mortality while waiting for, or shortly after, the start of second-line ART. Because of the operational challenges to obtain a viral load in our setting (as highlighted in figure 1), it may be appropriate to switch patients who fulfill the immunological and/or clinical WHO-criteria and have a score≥5 immediately to a second-line regimen without doing a confirmatory VL test. However, given that even those with a score<5 have a reasonable chance of failure (40%) the score cannot be used to exclude viral failure in this subgroup of patients.

This study has several limitations. First, we only measured one viral load per patient. A paper by Castelnuovo et al. showed that a majority of patients who have intermediate or high viremia achieve viral suppression at a second measurement [12]. Saag and colleagues report from a trial in Zambia that nearly half of patients achieved viral suppression at second measurement [13]. The overall failure rate may thus be overestimated in our study. A second limitation is that the applied score still relies on CD4-counts and is therefore not applicable in settings without access to CD4-count measurement. However, Marinucci et al. showed in an assessment in seven African countries that by 2010 already two thirds of rural ART-clinics had access to CD4-count on site [14]. Like the health centers in Lesotho, many other clinics that do not have CD4-counters on site may have a system of regular transport of blood samples to the referral hospital for analysis. A third limitation is that the patient population included may not be fully representative of all patients possibly failing first-line therapy. The study suffers from operational challenges inherent to working in low-resource rural settings. Only 92 (69%) out of 134 eligible patients could be included in the study, as for the others no VL-result could be obtained. Patients have to travel long distances to the health facilities and may be reluctant to come back for a blood test, especially when sick. This demonstrates the need to define criteria that allow switching patients with high probability of viral failure to second-line without prior confirmation through VL testing. Finally, in this rural setting some patients may not have a regularly documented CD4-count while on ART and ART-nurses may have overlooked some clinical WHO stage 4 conditions. Thus the overall number of patients fulfilling clinical and/or immunological WHO failure criteria in the study area may be considerably higher than the 134 patients that were identified during the study period.

Future studies on scoring systems in low-resource settings should look at criteria that do not depend on previous CD4 counts. Our study only looked at the PPV of the score≥5 in addition to WHO criteria. Looking at the individual PPV of the criteria that build the score, the strongest predictors are VAS<95%, a new onset papular pruritic eruption and a CD4-count <100 cells/µl after ≥12 months on ART (Table 3). As in some settings it may be difficult to have all data available to use the score (previous CD4-counts and previous hemoglobin levels), using these criteria alone as additional check before switching patients who fulfill immunological or clinical WHO-criteria to second-line would already increase the PPV for viral failure substantially.

The CD4-cell count alone, as it is used in the WHO definition of immunological failure, has shown limited accuracy in predicting viral failure [15], [16] and has led to misclassification and unnecessary as well as delayed switch to second-line ART [6]–[8], [17]–[19]. Because of the limited accuracy of WHO criteria, confirmation of treatment failure through VL testing has been recommended for all patients [20]. In our study, the PPV of WHO immunological criteria was higher than in previous studies [4], but a PPV of 44% for a VL≥5000 copies (Table 3) is still too low to justify switch to second-line ART without prior confirmation by VL-testing. However, to await a VL result for all patients presenting with failure is difficult in rural settings with limited resources and may result in delayed switching and high mortality. Several studies indicate that use of other laboratory and clinical information, such as adherence or changes in hemoglobin may improve the predictive value [9], [21]. In a South African cohort, Egger and colleagues have recently shown the usefulness of risk-charts that take into account CD4-trajectories over time on ART [22]. However, up to now, none has shown enough accuracy to replace VL testing. As long as cheap and simple to use tests for VL measurements are lacking, there is an urgent need to define clinical criteria that help to narrow down the population that needs VL testing.

### Conclusions

A clinical score≥5 in patients fulfilling the clinical and/or immunological WHO treatment failure criteria results in a high probability of viral failure. For these patients, especially in settings with limited access to VL-testing, we may consider switching patients to second-line treatment without prior VL-confirmation. This approach may save money and valuable time for the patient, thereby reducing mortality and lost-to follow-up. However, a lower score cannot be used to exclude viral failure. These patients require a confirmatory VL measurement prior to switch to second-line.
